# Demographic collapse and low genetic diversity of the Irrawaddy dolphin population inhabiting the Mekong River

**DOI:** 10.1371/journal.pone.0189200

**Published:** 2018-01-03

**Authors:** Michael Krützen, Isabel Beasley, Corinne Y. Ackermann, Dietmar Lieckfeldt, Arne Ludwig, Gerard E. Ryan, Lars Bejder, Guido J. Parra, Rebekka Wolfensberger, Peter B. S. Spencer

**Affiliations:** 1 Evolutionary Genetics Group, Department of Anthropology, University of Zurich, Zurich, Switzerland; 2 College of Science and Engineering, James Cook University, Townsville, Australia; 3 Department of Evolutionary Genetics, Leibniz Institute for Zoo and Wildlife Research, Berlin, Germany; 4 Centre of Excellence for Environmental Decisions, School of Biosciences, University of Melbourne, Victoria, Australia; 5 Cetacean Research Unit, School of Veterinary and Life Sciences, Murdoch University, Murdoch, Australia; 6 Cetacean Ecology, Behaviour and Evolution Lab, School of Biological Sciences, Flinders University, Adelaide, Australia; 7 School of Veterinary and Life Sciences, Murdoch University, Murdoch, Australia; National Cheng Kung University, TAIWAN

## Abstract

In threatened wildlife populations, it is important to determine whether observed low genetic diversity may be due to recent anthropogenic pressure or the consequence of historic events. Historical size of the Irrawaddy dolphin (*Orcaella brevirostris*) population inhabiting the Mekong River is unknown and there is significant concern for long-term survival of the remaining population as a result of low abundance, slow reproduction rate, high neonatal mortality, and continuing anthropogenic threats. We investigated population structure and reconstructed the demographic history based on 60 Irrawaddy dolphins samples collected between 2001 and 2009. The phylogenetic analysis indicated reciprocal monophyly of Mekong River *Orcaella* haplotypes with respect to haplotypes from other populations, suggesting long-standing isolation of the Mekong dolphin population from other *Orcaella* populations. We found that at least 85% of all individuals in the two main study areas: Kratie and Stung Treng, bore the same mitochondrial haplotype. Out of the 21 microsatellite loci tested, only ten were polymorphic and exhibited very low levels of genetic diversity. Both individual and frequency-based approaches suggest very low and non-significant genetic differentiation of the Mekong dolphin population. Evidence for recent bottlenecks was equivocal. Some results suggested a recent exponential decline in the Mekong dolphin population, with the current size being only 5.2% of the ancestral population. In order for the Mekong dolphin population to have any potential for long-term survival, it is imperative that management priorities focus on preventing any further population fragmentation or genetic loss, reducing or eliminating anthropogenic threats, and promoting connectivity between all subpopulations.

## Introduction

Freshwater cetaceans are among the world’s most threatened mammal species resulting from impacts of multiple human stressors, including habitat loss and degradation, pollution, entanglements in fishing nets and direct killing for bait, traditional medicine purposes and meat and oil for consumption [1–3]. Of the 90 cetacean species worldwide, three species (i.e., Amazon river dolphin *Inia geoffrensis*, South Asian river dolphin *Platanista gangetica* and Yangtze river dolphin *Lipotes vexillifer*) only reside in freshwater habitats (‘obligate’ or ‘true’ river dolphins), whereas three species (Yangtze finless porpoise, *Neophocaena asiaorientalis ssp*. *asiaorientalis*, Irrawaddy dolphin, *Orcaella brevirostris* and tucuxi *Sotalia fluviatilis and S*. *guianensis*) have populations in both coastal and riverine environments (‘facultative’ river dolphins). All obligate and facultative river dolphin populations in Asia have suffered dramatic population declines and are listed as *Endangered* or *Critically Endangered* on the International Union for Conservation of Nature (IUCN) Red List of Threatened Species. Although the Yangtze River dolphin or *baiji* is currently listed as *Critically Endangered*, the species is considered functionally extinct [4–6], which illustrates the vulnerability of these small isolated river dolphin populations. Demographic and genetic data are urgently needed to understand the vulnerability of freshwater dolphins to growing anthropogenic pressures, and contribute information required to prevent further extinctions.

The Irrawaddy dolphin is a small delphinid distributed along Asia’s coastal, lacustrine and riverine waters from the north-western Bay of Bengal southeast to the east coast of Kalimantan, Borneo, and south to the Indonesian island of Sumatra [7]. Lacustrine populations occur in Chilika Lake (India [8]) and Songkhla Lake (Thailand [9]), and riverine populations occur in the Mahakam River (Kalimantan [10]), Ayeyarwardy River (Myanmar [11) and Mekong River (Cambodia and southern Lao People’s Democratic Republic [hereafter referred to as Laos] [12]). Although some coastal Irrawaddy dolphin populations number in the thousands (e.g., Bangladesh [13–14]), most, if not all, lacustrine and riverine populations are small (less than 100 individuals) and face eminent local extinction. Apart from the Chilika Lake population, all lacustrine and riverine populations are classified as *Critically Endangered* by the IUCN [15–18].

**Fig 1:**
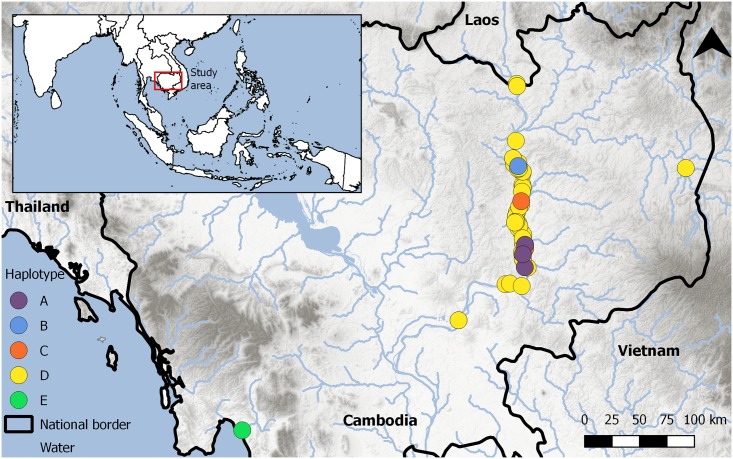
Sampling locations of individuals used in this study. Each coloured dot represents the approximate sampling location and mitochondrial DNA haplotype.

The Irrawaddy dolphin population that inhabits the Mekong River of southern Laos and northern Cambodia (hereafter referred to as the Mekong dolphin population) has been the subject of research since 1973 [19–22], with extensive effort occurring over the past 15 years [23–26]. Based on photo-identification studies, recent population estimates number less than 100 individuals [25,26,12]. The remaining Mekong dolphin population is now primarily restricted to the lower stretch of the Mekong River and some associated tributaries from Khone Falls (five kilometres north of the Laos/Cambodian border) south to Kratie in Cambodia (Fig 1).

There is immediate concern for the Mekong dolphin population’s long-term survival as a result of their low abundance, slow rate of reproduction and continued anthropogenic threats. The main anthropogenic threats are accidental catch in gillnet fisheries [27], un-regulated harassment caused by dolphin-watching vessels [28], direct deaths caused by illegal electric and dynamite fishing methods [27], contaminants and pollutants [29], and dam construction on the mainstream Mekong River in Laos and Cambodia causing significant environmental change and fragmentation of sub-populations [30–31]. An additional concern is a lack of recruitment into the population as a result of unusually high mortality of neonates and calves [25–26]. Little is understood of the genetics of this population, or how genetics studies may support its management. Some recent research suggests distinctness from nearby coastal populations, and signs of adaptation toward the freshwater habitat [32].

Based on photo-identification studies conducted from 2001–2007, there appear to be three main locations within the lower Mekong where Irrawaddy dolphins occur: ‘Cheuteal’, the transboundary pool below Khone Falls on the Laos/Cambodian border, ‘Stung Treng’, from the township of Stung Treng south to Tbong Klar pool, and ‘Koh Pidau/Kampi’, from below Tbong Klar pool south to Kampi Pool located north of the Kratie Township [26,33]. The Mekong dolphin population was estimated to be 93 individuals (95% confidence interval (CI) of 86–101) in 2007, based on research from 2004–2007 [26]. By 2010, the population was estimated to be 85 individuals (95% CI 78–91), based on data from 2007–2010 [25]. The most recent research, which extended the data gathered by [25] to April 2015, estimated 80 individuals, though the uncertainty around this estimate is larger (95% CI 64–100: [34]). Connectivity between the three locations is presumed to be limited, however potential wet season movements remain largely unknown [26].

In this paper, we aim to elucidate the conservation status of the Irrawaddy dolphin population inhabiting the Mekong River by: (a) investigating Irrawaddy dolphin genetic samples from Cambodia in a phylogenetic context; (b) analysing the potential presence and extent of population structure among Irrawaddy dolphins in the Mekong River; and (c) determining demographic parameters of the Irrawaddy dolphin population in the Mekong River.

## Materials and methods

### Study area

The Mekong dolphin population inhabits a 190 km section of the Cambodian Mekong River, from Kratie to Khone Falls on the Laos/Cambodian border (Fig 1). This river stretch consists of a variety of habitat types, ranging from deep pools (up to 90m) and shallow riffle habitat (i.e. short, relatively shallow and coarse-bedded length of stream over which the stream flows at slower velocity but a higher turbulence than a pool), with numerous islands that separate sections of the river into extensive channels of varying depth and width [15].

### Sampling and laboratory procedures

Samples were obtained from the Cambodian cetacean carcass recovery program, which has been operational since 2001 [27,24,33,35]. From 2000–2009, tissue and teeth samples were collected from 67 carcasses, comprising 29 adults (10 females, 8 males, 11 unknown), 35 calves (15 females, 14 males, 6 unknown), two juveniles (both females) and one foetus of unknown sex. Tissue samples were stored in 20% dimethyl sulfoxide (DMSO; [36]) at room temperature in the field and at -20°C upon arrival in the laboratory. Based on sampling locality and DNA quality, individuals sampled in the carcass recovery program and used in this study were assigned to one of the following three sites: Cheuteal (n = 2), Stung Treng (n = 12) Kratie (n = 42) (Fig 1). In addition to these individuals, two individuals were recovered from 100km south of Kratie, near Kampong Cham (added to ‘Kratie’ individuals), one individual was recovered from upstream the Sekong River, Mondulkiri Province (added to ‘Stung Teng’ individuals), and one individual was recovered from Sre Ambel, coastal Cambodia (Fig 1, SI Table 1).

We performed DNA extractions using the Gentra Puregene DNA Purification Kit/DNeasy^®^ Blood and Tissue Kit (Qiagen, Hilden) according to manufacturer’s instructions. For mitochondrial DNA, we amplified a segment of 384-bp containing the hyper-variable region I of the control region by means of the polymerase chain reaction (PCR), using primers dlp1.5 and dlp5 [37]. The PCR products were cleaned using a purification column (Quiagen) according to manufacturer’s instructions. We then sequenced PCR products with the ABI BigDye^®^ Terminator v3.1 Cycle Sequencing Kit (Applied Biosystems), using the forward primer dlp1.5, followed by analysis on an ABI 3730 DNA Analyser sequencer system (Applied Biosystems). Resulting sequences were edited with Sequencing Analysis, version 5.2 (Applied Biosystems). The alignment was carried out manually using the software LaserGene, version 6 (DNASTAR).

We also amplified the same samples using 21 autosomal microsatellites, which had previously shown to be highly polymorphic in different cetacean species: MK3, MK5, MK6, MK9 [38]; 66, 87,91, 98, 105, 108, 111, 117, 128, 138, 141, 142, 153, 162, F10 [39]; D22 [40]; and KWM12 [41]. PCR products were run on an ABI 3730 DNA Analyser. We measured fragment sizes using GeneMapper, version 4.0 (Applied Biosystems) software.

Given the poor quality of the samples, some of which originated from exhumed carcasses in a humid tropical environment, we were concerned about undue influences that allelic dropout could have on our analyses. Thus, we introduced rigid quality control measures to obtain high quality genotypes. First, we amplified each locus at least twice and independently. For individuals that appeared to be homozygous after this procedure, we carried out an additional three, independent PCRs to confirm homozygosity. Second, we also generated genotypes from six individuals independently to estimate genotyping error rates for each locus.

### Phylogenetic analysis

To assess phylogenetic relationships among dolphins from the Mekong River and other taxa of the genus *Orcaella*, we carried out a phylogenetic analysis using a Bayesian framework. Mitochondrial DNA sequences were obtained from GenBank from *Orcaella brevirostris* from India, the Philippines, Indonesia, Thailand, and the Cambodian coast, as well as from Australian snubfin dolphin *Orcealla heinsohni* from Australia [42]. As outgroups, we used mtDNA sequences from *Orcinus orca* and *Steno bredanensis*. We performed the analysis using program MrBayes version 3.2.2 [43–44]. Instead of selecting a substitution model, we sampled across the general time reversible (GTR) model space in the Bayesian Markov Chain Monte Carlo (MCMC) analysis itself, obviating the need to test for models *a priori*. We ran 20 x 10^6^ generations with a sample frequency of 10,000, discarding the first 25% of saved trees. Convergence of runs was determined by tools built into MrBayes, *i*.*e*. standard deviations of split frequencies below 0.01.

### Population genetic analyses

#### Mitochondrial DNA

Using mitochondrial DNA, we assessed genetic diversity within sampling localities by calculating haplotype and nucleotide diversity using Arlequin version 3.5.1.3 [45–46]. To test the degree of population differentiation among sampling localities, we calculated *ɸ*_ST_ values using Arlequin and applied a Tamura and Nei [47] with a γ-correction of 0.144, as computed by JModeltest version 2.1.3 [48]. Significance was obtained by performing 10,000 permutations.

#### Autosomal microsatellite data

The software package Structure version 2.3.3 [49] was used to determine the genetic structure and number of genetic clusters in our dataset. The Structure algorithm divides sampled individuals into a number of clusters (*K*) independent of locality information and by minimizing deviations from Hardy–Weinberg and linkage equilibrium in each cluster. The program uses a MCMC procedure to estimate P(X|*K*), the posterior probability that the data fit the hypothesis of *K* clusters. For all analyses, the length of the burn-in period was set to 10^5^, followed by 10^6^ MCMC steps. For each *K*, the analysis was run ten times. We chose the Locprior model, which improves clustering when the signal is weak without spuriously inferring structure, if absent [50].

Using data generated from the autosomal microsatellites, we estimated genetic variation within and genetic differentiation between sampling sites. Prior to this, we tested for significant deviations from Hardy-Weinberg equilibrium (HWE) expectations for each locus and population, as well as the presence of linkage disequilibrium (LD) among all loci, using the software Genepop, version 4.2.1 [51–52]. A Bonferroni correction for multiple tests was subsequently applied for each population and locus [53].

Genetic variation within sampling locations was determined by estimating the number of alleles per locus (*N*_a_), number of effective alleles per locus (*N*_e_), and calculating unbiased levels of expected (u*H*_e_) and observed heterozygosity (*H*_o_), using the software GenAlEx, version 6.5 [54]. To estimate genetic differentiation between sampling locations, we calculated Weir and Cockerham’s θ [55] in Genepop and D_est_ [56–57] using Smogd, version 1.2.5 [58]. In addition, the number of private alleles per population was calculated as described in [59] with GenAlEx.

### Demographic modelling

We detected change in the effective population size using three approaches. The first two used summary statistics to detect a change in allele frequencies while the last one used a full likelihood Bayesian method that also allowed us to detect, quantify and date the change in size. The first approach was implemented in Arlequin and developed by [60]. This approach may identify bottlenecks if they had lasted several generations, the pre-bottleneck θ was large, and the population had made a demographic recovery. The approach takes advantage of the fact that reductions in population size can be used to estimate the mean ratio of the number of alleles to the range in allele size [60] using a statistic *M*k. The value of *M*k has been shown to decrease when a population is reduced in size over recent times (<10 generations) and as such, the statistic *M*k can distinguish between populations that have been recently reduced in size to those that have been small for a long time [60]. An expected distribution for *M*k under equilibrium conditions was generated using simulations. The critical value is at the lower 95th percentile of this distribution, and a bottleneck identified when a value of *M*k is lower than this critical value [60].

The second approach was developed by [61], and inferred genetic bottlenecks as a result of an excess of heterozygotes under a stepwise mutation model. We employed the software Bottleneck, version 1.2 [62] to test for distortion of allele frequency distributions as a result of rare alleles being more likely to be lost during a bottleneck than common alleles. This test for a genetic bottleneck is appropriate for populations that have been reduced very recently with little severity, and a small pre-bottleneck value of θ [63]. We tested three models of microsatellite mutation (Infinite Allele Model—IAM, Two-Step Model—TPM, and the Stepwise Mutation Model—SMM). For the TPM, we assumed that single step mutations account for 90% of all mutation events, and a variance among multiple steps of 12, as suggested by Piry et al. (1999). Due to the relatively small number of loci analysed (n = 10), we used a Wilcoxon sign-rank test to estimate significance [62]. Despite its use, we believe that with 10 polymorphic loci, low heterozygosity, and a significant reduction in pre/post bottleneck size (data from our results; see [64]), there will be only very low power to accumulate a detectable excess.

We also employed a Bayesian approach developed by [65] for detecting changes in population size. This approach makes use of coalescent theory to create *posterior* distributions from different *prior* demographic parameters, given the observed allele frequency distributions at the sampled loci. The main assumptions of the approach are a population of constant size of *N*_1_, followed by an increase (or decrease) *t*_*a*_ generations ago to the current population size of *N*_0_. Changes are assumed to be either linear or exponential at a rate of *θ* = 2*N*_0_*μ* (*μ* = mutation rate) under a stepwise mutation model.

First, we employed a Bayesian coalescent-based MCMC approach implemented in the software MsVar version 0.4.2 to estimate posterior probabilities of the size of population size change (*r* = *N*_0_/*N*_1_), the timing of when the change occurred (*t*_f_ = *t*_a_/*N*_0_—a measure scaled by *N*_0_), and the scaled mutation rate θ. We ran five independent chains with different starting demographic histories, using wide uniform priors of between -5 and 5 (on a log_10_-scale) for *θ*, *r* and *t*_*f*_.

With the software MsVar version 1.3, we also used a method by [66] to quantify the effective population size of the current (*N*_0_) and the ancestral (*N*_1_) population, as well as the time (*T*) since the population size changed. In this model, the prior distributions of *θ*, *N*_0_, and *T* are assumed to be log-normal. As such, we used wide uninformative priors, multiple runs (at least three independent runs as a test of convergence, with differing starting seeds) and varying hyperprior parameters. MsVar was run for 180 simulations with 10^9^ iterations each using a cluster array at http://www.ivec.org/. Current population size was set to N_0_ = 101 [26]. We used the Irrawaddy dolphin generation time of 20 years based on [67].

Outputs for MSVAR were interpreted using purpose-written scripts in R v2.10.1 (R Development Core Team 2009). We discarded the initial 10% of sampled points for each run and checked convergence of chains visually and formally using the Gelman-Rubin statistic [67–69], with R library: boa [70]) for Log_10_ (*θ*, *r* and *t*_*f*_). It has been shown that a point estimate (and 95% quartile) of <1.2 is indicative of satisfactory convergence [71]. Approximate posterior densities for 0.1, 0.5 and 0.05 (highest posterior density limits; HPD) were calculated and graphically represented (R library: boa, hdrcde and Locfit).

### Research approvals and ethics

This research was approved by the Centre for Ethics at the University of Zurich and James Cook University Ethics Committee. All fieldwork was approved by the Royal Government of Cambodia, with the relevant CITES Permits obtained for export of samples.

## Results

**Fig 2:**
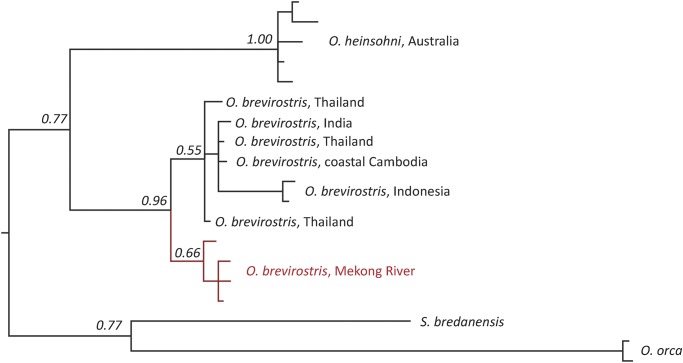
Phylogenetic relationships of the genus *Orcaella* based on 384bp of the hyper-variable region I of mitochondrial DNA. Numbers indicate Bayesian posterior probability values for each clade.

The phylogenetic analysis indicates reciprocal monophyly of the Mekong dolphin population haplotypes with respect to haplotypes from other populations, suggesting a long-standing isolation of the Mekong dolphin population from other *Orcaella* populations (Fig 2). *Posterior* probability values support a clear split between *O*. *brevirostris* from the Mekong River and populations from other areas, including coastal Cambodia, approximately 700km from the Mekong Delta.

Mitochondrial haplotype and nucleotide diversity indices were estimated from 55 individuals, from which we were able to obtain mtDNA sequence data. Based on their origin, individuals for this analysis represented three sampling regions: Kratie (n = 42), Stung Treng (n = 12) and Cheuteal (n = 1). In the Kratie and Stung Treng regions, haplotype D was the most common, with at least 85% of all individuals in each region bearing the same haplotype (Table 1). There was only data for one individual from the Cheuteal region, which also showed the most common haplotype D. The low haplotype diversity was reflected in the nucleotide diversity at each site, which was extremely low (Table 1). The AMOVA revealed a complete lack of genetic structure for mitochondrial DNA within our dataset (Table 2). Only a non-significant (*P =* 0.32), very small portion (1.73%) of the total genetic variation was attributed to genetic differences between the sampling sites.

**Table 1:** Haplotype distribution and diversity at three sampling sites.

Haplotype	Kratie (*n* = 42)	Stung Treng (*n* = 12)	Cheuteal (*n* = 1)
**A**	3 (7.1%)		
**B**		1 (7.7%)	
**C**	2 (4.8%)		
**D**	37 (88.1%)	12 (92.3%)	1 (100.0%)
			
**Haplotype diversity *h***	0.26 (± 0.08)	0.17 (± 0.13)	n/a
**Nucleotide diversity *π***	0.0010 (± 0.001)	0.0004 (± 0.0007)	n/a
**Genetic differentiation *ɸ*_ST_**	0.017 (*P =* 0.32)		n/a

**Table 2:** Analysis of molecular variance using Kratie and Stung Treng as sampling locations.

Source of variation	Degrees of freedom	Sum of squares	Variance components	Percentage of variation
**Among Locations**	1	0.262	0.00347	1.73
**Within Locations**	52	10.252	0.19716	98.27
**Total**	53	10.514	0.20063	

Given the challenging sample quality, we were able to generate microsatellite genotypes for 44 individuals. Out of the 21 loci tested, eleven were monomorphic (Table 3). Over all 21 loci, expected heterozygosity values were extremely low (Kratie: u*H*_e_21 = 0.22, Stung Treng u*H*_e_21 = 0.19, combined: u*H*_e_21 = 0.21). The number of private alleles was higher in Kratie (n = 6) than in Stung Treng (n = 1), although this was expected given the almost threefold difference in sample size between the two.

**Table 3:** Genetic diversity estimates based on 21 scored microsatellite loci. Eleven loci were monomorphic, thus, summary statistics are also presented for the ten polymorphic loci. *N =* mean number of samples/locus; *N*_a_ = average number of alleles/locus; *N*_e_ = mean number of effective alleles/locus; *H*_o_ = mean observed heterozygosity; u*H*_e_ = mean unbiased expected heterozygosity; S.E. = standard error. Numerical indices in the top refer to number of microsatellite loci on which summary statistics are based.

Sampling site		*N*21	*N*_a_21	*N*_e_21	*H*_o_21	u*H*_e_21	*N*10	*N*_a_10	*N*_e_10	*H*_o_10	u*H*_e_10
**Kratie (*n* = 32)**	Mean	21.33	2.10	1.45	0.21	0.22	26.80	3.30	1.95	0.43	0.46
	S.E.	1.52	0.30	0.14	0.05	0.06	2.09	0.34	0.19	0.05	0.05
**Stung Treng (*n* = 12)**	Mean	8.33	1.86	1.33	0.17	0.19	9.90	2.80	1.69	0.35	0.46
	S.E.	0.43	0.24	0.10	0.05	0.05	0.53	0.29	0.14	0.07	0.05
**Combined**	Mean	29.67	2.14	1.43	0.19	0.21	36.70	3.40	1.91	0.41	0.45
	S.E.	1.90	0.32	0.13	0.05	0.05	2.51	0.40	0.18	0.05	0.04

Genetic differentiation between all sampling localities was low. Both *F*_ST_ and Jost’s *D* were small (*F*_ST_ = 0.044, *D =* 0.004) and non-significant. The Structure analysis corroborated these findings, as we did not detect any population structure (Log-likelihood values for five independent runs for each *K* indicated that *K =* 1 had the highest probability) even when using a model with the highest chance to detect weak structure. Combining these results and the lack of differentiation found mitochondrial DNA, we considered both sampling locations as one population for subsequent analyses of demographic change.

Evidence about recent bottlenecks was equivocal. Estimates of *M*k (the mean ratio over all loci of the number of alleles over the range in allele size) were low, ranging from 0.26 to 0.67 (*M*k_10 loci_ *=* 0.39, s.d. = 0.13). However, no values were significantly lower than those expected by chance (P>0.05), even under wide *θ* starting–values and current effective population size spanning 100 to 10,000 dolphins. In the Bottleneck analysis, we did not detect a deviation from a normal L-shaped distribution of alleles, and no significant bottleneck using a Wilcoxon rank sum test under a SMM (*P* = 0.55), TPM (*P* = 1.00), or IAM (*P =* 0.19) model of mutation, as predicted (SI Table 2).

**Fig 3:**
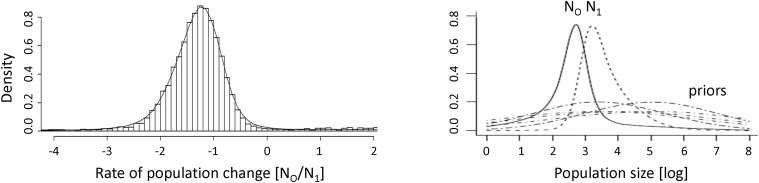
Posterior distributions of rates of population change [log_10_(*r*)] as determined with MSVar 0.4.2 (left) and changes in population size as determined with MSVar 1.3 (right).

Posterior distributions of the rate of population change in the Mekong dolphin population, as calculated with MsVar, version 0.4.2, suggested that there has been a recent exponential decline in the population (Fig 3). All models invoking a linear change of population size did not converge, as indicated by the Gelman statistic [65], while all runs with exponential changes converged and were remarkably consistent, despite a wide range of prior parameters (SI Table 3). The median log_10_(*r*) ratio of current (*N*_0_) to ancestral (*N*_1_) effective population sizes was -1.20 (95% HDR -2.85–0.18) across all runs (SI Table 4), indicating that a decline in the effective size of all populations of about one order of magnitude and no support for stability (log_10_(*r*) = 0) or expansion (log_10_(*r*) > 0). The decline occurred fairly recently, as median log_10_*t*_f_ (2.2) was small.

Results from the MsVar, version 1.3 runs, using the method by [66] to quantify changes in the effective populations size, suggested an exponential decline of the Mekong dolphin population of about 19 times its original source size (Fig 3, SI Table 4). Similar to simulations from MsVar 0.4.2, none of the linear runs converged, as indicated by the Gelman statistic [65]. The *posterior* distributions for *N*_0_ and *N*_1_ showed only a moderate overlap, confirming the occurrence of a population decline. Current *N*_0_ (median log *N*_0_ = 1.89, 95% HDR = 0.11–3.15) was more than an order of magnitude smaller than *N*_1_ (median log *N*_1_ = 3.17, 95% HDR = 1.57–4.91). These data suggest that the historic population size was at least 19 times larger than the current one, or that the current effective population size is only 5.2% of that of the ancestral population. Assuming a generation time of 20 years for Mekong River *Orcaella*, the decline might have started ca. 145,000 years ago (95% HDR 159–1,584,893 years).

## Discussion

The Irrawaddy dolphin population occupying the Mekong River is small, declining, isolated, and potentially suffering from inbreeding and loss of genetic diversity. It is apparent that this population is in serious threat of extirpation in the near future. Of the three riverine populations of Irrawaddy dolphins, the population that inhabits the Mekong River is likely the largest and certainly the most extensively studied. Thus, the capacity for this population to recover, and our ability to assist with its recovery, are potentially greater than the other riverine populations of Irrawaddy dolphins.

Our phylogenetic analysis indicated reciprocal monophyly of Mekong dolphin population haplotypes with respect to haplotypes from other populations, suggesting a long-standing isolation of the Mekong dolphin population from other Irrawaddy dolphin populations, including coastal Cambodia. However, further Irrawaddy dolphin genetic samples, particularly from coastal areas near the Vietnamese Mekong Delta and other riverine populations, are required in order to elucidate whether sub-species level designation is justified. Moreover, to increase resolution and support, data from full mitochondrial genomes should be generated. Regardless of the need for further samples, our limited dataset indicates that the Mekong dolphin population conservation efforts should be expanded and intensified as a matter of priority, to reduce the local extinction probability of this potentially unique sub-species.

Our data suggest that the different sub-populations along the Mekong River should be treated as a panmictic population. However, even at a combined level, genetic variability is extremely low. The causes for this could be manifold, and unfortunately our data are insufficient to clearly disentangle different scenarios of how the low genetic diversity originated. Given the limited occurrence of Mekong River Irrawaddy dolphins and their genetic isolation, a potential explanation is that the founding population is fairly young and had a small effective population size to start with. Moreover, given the slow generation time of 20 years [67], this population might have never accumulated a substantial amount of genetic diversity via mutation and/or immigration.

Being part of the Sunda shelf, the Mekong River system was subjected to repeated geological transformations during multiple glacial periods during the Pleistocene (between 2.58 Mya and 11.8 kya: [72,42,73–74]). Consecutive cycles of cooling and warming resulted in different sea levels, so that land-masses and river systems fluctuated greatly in size and geographic extent over time [75]. These changes in length and depth of different river stretches would certainly have had consequences for the connectivity of different river stretches to one another, as well as for the coastal area and deep water pools within the upper Cambodian Mekong River. Based on these complex geological features and the broad confidence intervals of our demographic analyses, it is unfortunately not possible to determine whether the current low genetic diversity observed in the Mekong dolphin population is an ancient effect.

In recent times, a significant loss of individuals was probably caused by the intensive bombing of Laos, Cambodia and Vietnam during wars from the 1950s to 1970s, as well as intentional killings of dolphins in Tonle Sap Lake for their oil during the same period [21–22,76]. There are no estimates on the number of dolphins killed, but the area along the eastern Cambodian and Vietnamese border, and especially the Mekong River, was considered a potential supply route of the Vietcong, and struck by more than 2.7 million tons of explosives between 1964 and 1975 [76]. However, given the slow life history of Irrawaddy dolphins, the observed extremely low current genetic diversity cannot be entirely explained by the substantial number of dolphins killed during the 1960s and 1970s.

### Conservation considerations

To ensure both the long-term persistence and evolutionary potential of the Mekong dolphin population, drastic management measures are required immediately by the relevant Governments, conservation agencies and local Cambodian and Laos communities.

Since the 1970’s, dolphins have been shot for target practice, accidentally by-caught in subsistence fisheries, and have been directly killed through dynamite and electric fishing [20–22,33,77]. Recent anthropogenic threats include potential contaminants from gold-mining and agricultural run-off [78] and potential harassment from dolphin-watching vessels [28]. The high newborn mortality is an additional concern, with no apparent cause being discovered despite extensive investigations [79,24]. Since 2009, an *ad hoc* team of international scientists have been working with the Cambodian Government and WWF-Cambodia to co-ordinate and develop Mekong dolphin conservation efforts, with the landmark ‘*Kratie Declaration on the Conservation of the Mekong River Irrawaddy dolphins*’ being finalized in 2012 [79]. Although progress has been made with patrolling activities and the refinement of protocols to investigate mortality, entanglement in gillnets is reportedly one of the most critical and immediate threat to survival of dolphins in the Mekong River [80–81,26]

The construction and operation of the Don Sahong hydropower dam on the Laos/Cambodian border is also a major concern, particularly to the small sub-population of dolphins (now numbering only three individuals: [34] that inhabit the Laos/Cambodian border region of Cheuteal [81,31]. Construction and operation of the Don Sahong dam would almost certainly cause local extirpation of the already small Cheuteal sub-population as a result of increased noise (particularly from explosives used during the construction phase), increased boat traffic, and reduced prey from blockage of a major fish passage during the dry season [31,82]. The proposed Sambor Dam, which would be constructed across the mainstream Mekong River north of Kratie, is of significant concern due to its likelihood of fragmenting the remaining Mekong dolphin population and potential environmental impacts [83,84,85]. Construction of these dams increases extinction risk of the entire Mekong dolphin population [31,82]. Fragmentation of river dolphin meta-populations by dams and irrigation barrages is an increasing problem for the long-term survival of river dolphin populations world-wide [11,86–87], where loss of connectivity, reduced water and prey availability, and close proximity to anthropogenic activities accentuates the difficulties of river dolphin conservation [28].

Gene flow among small fragmented populations is critical for maintaining genetic diversity, and therefore the evolutionary potential of a species [88]. When gene flow is restricted, low abundance sub-populations, or population fragments, become increasingly vulnerable to loss of genetic variation via genetic drift, accumulation of deleterious mutations, inbreeding depression, and inability to adapt to change [89], all of which are correlated with the risk of extinction. Alleviating inbreeding depression by transferring new breeding stock into severely inbred and isolated populations (‘*genetic rescue*’), as carried out in panthers [90,91], prairie chickens [92], adders [93], and ibex [94], are not considered a viable options for cetacean populations.

Given that ‘genetic rescue’ is not feasible for the Mekong dolphin population, or cetaceans in general, management efforts should focus on effectively managing all locations where Mekong dolphins occur (i.e. Cheuteal, Stung Treng, Koh Pdao, and Kratie); with the Cheuteal population being a high priority, given its small size of three individuals and isolated location 70km north of the Steng Treng sub-population. Important considerations are ensuring continued connectivity between sub-populations as well as reduction of anthropogenic threats, such as accidental entanglement in gillnets and boat-based dolphin watching tourism by changing to land-based tourism. Population monitoring is an essential component of any species recovery program, where photo-identification to assess trends in abundance, and carcass recovery programs to monitor mortality rates and causes, is required to monitor the remaining population and changes in response to management initiatives [25,26].

Captive breeding and reintroduction have not played a major role in the conservation of small cetaceans [95]. At present, the prospects of establishing an Irrawaddy dolphin captive breeding and reintroduction program are not recognised as viable options because of the inherent risks associated with the capture process, a lack of reliable care and husbandry techniques for Irrawaddy dolphins, the known poor survival rate for Irrawaddy dolphins in captivity [96], and the lack of substantial Cambodian/Vietnamese coastal Irrawaddy dolphin populations from which a large number of translocatable individuals could be taken. There is also a lack of knowledge about possible local adaptions required for the translocated individuals, such as prey requirements, site fidelity or social structure [33]. Importantly, as discussed in [91], there is little justification to ‘*rescue*’ small isolated populations if known threats are continuing.

The potential for extinction is now a reality for the Mekong dolphin population, particularly with construction of the Don Sahong Dam, and potential construction of the Sambor Dam. This study suggests a long-standing isolation of the Mekong dolphin population from other *Orcaella* populations, with the remaining population now experiencing low genetic diversity. In order for the Mekong dolphin population to have any potential for long-term survival, it is imperative that any further population fragmentation or genetic loss is prevented, anthropogenic threats are reduced, and continued connectivity between all regions is a high management priority.

## Supporting information

S1 Fig Permission to publish Fig 1 under a CC BY license. (XLSX)
